# Analysis of Psychological and Gut Microbiome Characteristics in Patients With Non-erosive Reflux Disease

**DOI:** 10.3389/fpsyt.2021.741049

**Published:** 2022-01-13

**Authors:** Fan Yang, Xin-hui Xie, Xi Li, Hui-na Liao, Bing Zou

**Affiliations:** ^1^Department of Gastroenterology, Peking University Shenzhen Hospital, Shenzhen, China; ^2^Department of Psychiatry, Shenzhen Mental Health Center, Shenzhen Kangning Hospital, Shenzhen, China; ^3^Brain Function and Psychosomatic Medicine Institute, Second People's Hospital of Huizhou, Huizhou, China; ^4^Center of Acute Psychiatry Service, Second People's Hospital of Huizhou, Huizhou, China

**Keywords:** non-erosive reflux disease, anxiety, somatoform disorders, gut microbiome, microbiome-brain-gut axis

## Abstract

**Objective:** To assess the correlation between the incidence of non-erosive reflux disease (NERD) and psychological factors, especially somatoform disorders. To investigate the characteristics of gut microbiome in NERD patients.

**Methods:** We enrolled 24 NERD patients and 24 healthy controls. All patients were evaluated via GerdQ, SOMS-7, SAS, HAMA, and HAMD. Fecal samples were collected and 16S rRNA sequencing was performed to evaluate the gut microbiome composition.

**Results:** The main symptoms of the NERD patients were regurgitation (87.5%), belching (66.7%), pharyngeal discomfort (50%), and heartburn (37.5%). The average score of GerdQ was 13.42 ± 3.41. In 15 patients (62.5%), the total score of the last two items was <3 points, while the average score of 24 patients was 3.63 ± 2.32. NERD patients with somatoform disorders accounted for 50%. There were 17 patients without anxiety, 6 patients with mild anxiety (25%), 1 patient with moderate anxiety (4.2%), and no patient with severe anxiety. There were 22 patients (91.7%) without depression, 2 patients (8.3%) with mild depression, and no patient with moderate or severe depression. The alpha diversity of NERD group was higher than HC, which showed significant difference (*P* < 0.05). The beta-diversity was significantly different between HC and NERD patients (*P* = 0.026), male and female patients (*P* = 0.009). The beta-diversity was also significantly different between male and female patients (*P* = 0.009). There were several bacteria with significant differences between HC and NERD group, and NERD patients with or without somatoform disorders, such as Firmicutes, TM7 were enriched in the NERD group compared with the healthy control group, while Bacteroidetes were enriched in the healthy controls.

**Conclusions:** NERD symptoms overlap with somatoform disorders. NERD symptoms have an impact on the daily life quality of patients. Some of them are accompanied by anxiety and depression of different degrees, and the two are significantly correlated. The diversity of gut microbiome in patients with NERD is significantly higher than healthy controls, which has its characteristics. The predominant bacteria in gut microbiome of patients with NERD are similar to the healthy population, with Firmicutes and Bacteroidetes as the main ones. The composition of gut microbiome in NERD patients with or without somatoform disorder is significantly different, which may be related to the interaction of microbiome-brain-gut axis.

## Introduction

Gastroesophageal reflux disease (GERD) is a common upper gastrointestinal disease in both Western countries and Asia, defined as a condition which develops when the reflux of stomach contents causes troublesome symptoms such as heartburn and regurgitation, and/or complications. Heartburn is defined as a burning sensation in the retrosternal area (behind the breastbone), while regurgitation is defined as the perception of flow of refluxed gastric content into the mouth or hypopharynx (1). As one subtype of GERD, non-erosive reflux disease (NERD) has been commonly defined as the presence of classic GERD symptoms caused by intraesophageally reflux (acidic or weakly acidic), in the absence of visible esophageal mucosal injury during upper endoscopy (2).

From precious studies, NERD has been reported in more than 50% of the Western population and the incidence of NERD has been increasing annually. In Asia, Rosaida and Goh have shown in a carefully studied prospective study that 65.5% of their patients had NERD (3, 4). Typical symptoms associated with NERD include heartburn and regurgitation, as well as extraesophageal manifestations such as nausea, chronic cough, asthma, and hoarseness that can affect the quality of their daily lives, including social functioning, sleeping, and work productivity (5, 6).

Many studies have revealed the relationship between described symptoms of NERD and emotional status. There is a large overlap in the symptomatic spectrum between NERD and somatoform disorders (7). A cross-sectional study revealed that anxiety and depression levels were significantly higher in subjects with GERD (notably in the NERD) than in controls (8). A case-control study also showed that mental factors (anxiety and depression) play important roles in the development of GERD, especially NERD (9). Most of NERD patients with nighttime heartburn have sleep disturbances. These subjects also had more depression, more anxiety, more severe gastrointestinal reflux disease symptoms, and higher prevalence of NERD (10, 11). Actually, psychotherapy combined with medication can relieve clinical symptoms and improve quality of life to varying degrees in patients with NERD (12). The factors that link NERD and psychology are not clear yet, but gut microbiome might play a role.

The numbers of bacteria in the Human Gut Microbiome are estimated to be close to equal to human cells (about 3.9 × 10^13^) (13). The complex bidirectional communication between the gut and the brain is finely orchestrated by different systems, including the endocrine, immune, autonomic, and enteric nervous systems (14). For example, in animal research conducted by Kelly et al., fecal microbiota transplantation from depressed patients to c-depleted rats can induce behavioral and physiological features of depression in the recipient animals, which suggests that the gut microbiome may play a causal role in the development of features of mental disorders (15). Stevens and Goel et al. got the results to support their hypothesis that anxiety and depressive disorders are associated with gut dysbiosis (16).

The underlying mechanisms associated with NERD and emotion were still unclear. Based on the above summary, we assumed that the gut microbiome may be an important part, so we designed this study to test this hypothesis.

## Materials and Methods

### Patient Enrollment and Sample Collection

From February 2019 to February 2020, 24 patients [7 males, 17 females, mean age (43 ± 11.0) years old] with NERD who admitted to the Departments of Gastroenterology and 24 healthy controls [7 males, 17 females, mean age (44 ± 9.6) years old] from Physical Examination Center in Peking University Shenzhen Hospital were enrolled in this study. The patients were further grouped into NERD without emotional disorders (*n* = 12) and NERD with somatoform disorders (*n* = 12). The study was approved by the ethics committees of Peking University Shenzhen Hospital, and all the patients signed the informed consent forms. All methods were carried out in accordance with relevant guidelines and regulations. The age, gender, and body mass index (BMI) of the healthy control group was matched with the group of NERD patients.

All participants provided their fecal samples. Stool samples were collected from fresh feces in the clean environment, put in sterile sampling tubes and stored at −80°C, and delivered to the laboratory of hcode Biological Technology company in Shenzhen.

### Inclusion and Exclusion Criteria of Cases

The NERD diagnostic criteria are as follows: (1) patients with typical heartburn, regurgitation, chest pain (distinguishable from ischemic cardiac pain), and other extraesophageal manifestations such as chronic cough, chronic laryngitis, and asthma (17); (2) patients meeting the previous conditions who also have GerdQ scores over 8 points despite gastroscopy indicating no esophageal mucosal damage or Barrett's esophagus (18). **Exclusion Criteria**: (1) peptic ulcer, tumors, pharyngeal organic diseases, and those with a history of digestive tract surgery; (2) neurological disease; (3) previous treatment for anxiety, depression, or other mental illness; (4) antibiotics, probiotics, immunosuppressants, and other drugs have been used in the past 2 weeks; (5) women during pregnancy or lactation; (6) disinclination to cooperate with the questionnaire or poor compliance.

The group of healthy controls: (1) without heartburn, regurgitation, and other symptoms of stomach discomfort; (2) past physical health, without chronic diseases; (3) voluntary participation in the study.

### Questionnaires

The general data of enrolled patients, including age, gender, main symptoms, and symptom duration were recorded. The presence of GERD symptoms and their severity and frequency were evaluated by certified gastroenterologists. These symptoms were verified with a language-specific version of international Gastroesophageal Reflux Disease Questionnaire (GerdQ), which consisted of 6 questions as follow: four positive questions (heartburn, regurgitation, sleep disturbance related to heartburn, and regurgitation and use of medications) and two negative questions (epigastric pain and nausea). Each item rated from 0 to 3 depending on the rate of symptoms over the previous week. GERD was detected with a total score value of 8 or more (19, 20). A total score value of 8 points or higher was considered positive for the presence of GERD, while it plus total score of 3 or more for the impact questions (sleep disturbance and use of medications) indicted GERD with impact on daily life.

### Evaluation of the State of Emotional Disorders

The Chinese version of new Somatoform Symptoms-7 (SOMS-7) asks for the existence and intensity of 53 somatoform symptoms to assess somatoform disorders. If female patients have 3 or more positive items and the total score ≥16, male patients have 2 or more positive items and the total score ≥15, somatic disorders can be indicated clinically (21, 22). The Self-Rating Anxiety Scale (SAS) is a questionnaire with 20 items used to assess anxiety over the past weeks. Each item is scored on a 4-point Likert scale: 1 (no or a little of the time), 2 (some of the time), 3 (good part of the time), and 4 (most of the time or all the time). The scores for all items were summed to obtain a crude score and then multiplied by 1.25 to obtain the standard score. Scores are categorized as no anxiety (below 49), mild anxiety (50–59), moderate anxiety (60–69), and severe anxiety (above 70) (23).

Hamilton Anxiety Rating Scale (HAMA) and Hamilton Depression Rating Scale (HAMD) were used to assess the severity of anxiety and depression respectively. The two scales were assessed by a professional psychologist and a gastroenterologist, and the two gave the final results of the consistency evaluation. Patients were scored according to the severity from 0 to 4. HAMA consists of 14 items [HAMA score: ≥7 points and <14 points indicates possible anxiety; ≥14 points and <21 points indicates definite anxiety; ≥21 points, certainly have significant anxiety]. HAMD contains 17 items yielding a maximum score of 52, with higher scores indicating greater depressive symptom severity [HAMD score: >7 points and ≤17 points indicates mild depression; >17 points and ≤24 points indicates moderate depression; >24 points indicates severe depression] (24, 25).

### Fecal Microbiome Analysis

The QIAamp Fast DNA Stool Mini Kit (Qiagen, Germany) was used to isolate total microbial DNA from stool with a procedure reported previously. To characterize the taxonomic profiles of the gut microbiome, the V3-V4 hypervariable region of the 16S rRNA gene was PCR amplified with dual-indexed PCR primers. The size and quality of the purified amplicons were checked with an Agilent 2100 Bioanalyzer (Agilent, USA). An equal amount of each amplicon was pooled and sequenced with Illumina MiSeq platform. The low quality and chimeric sequences were filtered via software USEARCH. The cluster operational taxonomic units (OTUs) were generated based on 97% nucleotide similarity (cluster_otus command). Greengene 13.5 reference database was employed for taxonomic assignment.

The gut microbiome alpha diversity was measured using the Shannon, Chao1, and Simpson indexes. The beta diversity among samples were calculated through Principal Coordinate Analysis (PCoA) to Bray-Curtis distance based on the OTU abundance. Statistical differences in alpha diversity and relative bacterial abundance were accessed by Wilcoxon rank-sum test for comparison of two groups, and one-way ANOVA followed by Tukey's test for comparison among three or more groups. Kyoto Encyclopedia of Genes and Genomes (KEGG) Ortholog functional profiles of microbial communities were predicted by Phylogenetic Investigation of Communities by Reconstruction of Unobserved States (PICRUSt), and the statistical significance was determined by Linear Discriminant Analysis (LDA) Effect Size (LEfSe) method (26, 27).

## Results

A total of 48 subjects were enrolled: **Group E**: 24 patients with NERD (The duration of illness ranged from 1 month to 7 years), **Group C**: 24 healthy controls (HC).

### Clinical Features of the Samples of 24 Patients

The GerdQ scores of the 24 patients were all more than 8 points (mean score 13.42 ± 3.34) and all of their gastroscopy indicated no mucosal damage, which illustrated that the 24 patients met the NERD clinical diagnostic criteria. In addition, 15 patients had sleep disturbance (were divided into SD group), and the other 9 patients were divided into group NSD (NERD patients without sleep disturbance). Meanwhile the total scores of the two impact questions were over 3, which indicated that NERD has affected their daily life.

The main symptoms of the NERD patients were regurgitation (87.5%), belching (66.7%), pharyngeal discomfort (50%), and heartburn (37.5%). Other symptoms, including abdominal pain (29.16%), abdominal distension (25%), nausea (20.83%), cough (8.33%), diarrhea (1.25%), were mentioned.

According to the results of Table 1, the 24 patients had varying severity of emotional disorders. Finally, we decided to divide them into group A (NERD patients with somatoform disorders) and group N (NERD patients without somatoform disorders); group F (Female patients) and group M (male patients); and group NSD (patients with non-sleep-disorder) and group SD (patients with sleep-disorder).

**Table 1:** Evaluation of the state of emotional disorders.

**Questionnaires**	**The severity of anxiety and depression (score)**	**Average score**	***N* (percentage)**
SAS	No anxiety (≤49)	17.46 ± 15.42	17(70.8%)
	Mild anxiety (50–59)		6(25%)
	Moderate anxiety (60–69)		1(4.2%)
	Severe anxiety (≥70)		0(0.0%)
HAMA	No anxiety (≤6)	9.33 ± 5.41	11(45.8%)
	Possible anxiety (≥7, <14)		9(37.5%)
	Definite anxiety (≥14, <21)		2(8.3%)
	Significant anxiety (≥21, <29)		2(8.3%)
	Severe anxiety (≥29)		0(0.0%)
HAMD	No anxiety (≤7)	2 ± 3.58	22(91.7%)
	Mild depression (>7, ≤17)		2(8.3%)
	Moderate depression (>17, ≤24)		0(0.0%)
	Severe depression (>24)		0(0.0%)
SOMS-7	Positive	17.46 ± 15.42	12(50.0%)
	Negative		12(50.0%)

### Alpha Diversity Analysis

**Figure 1:**
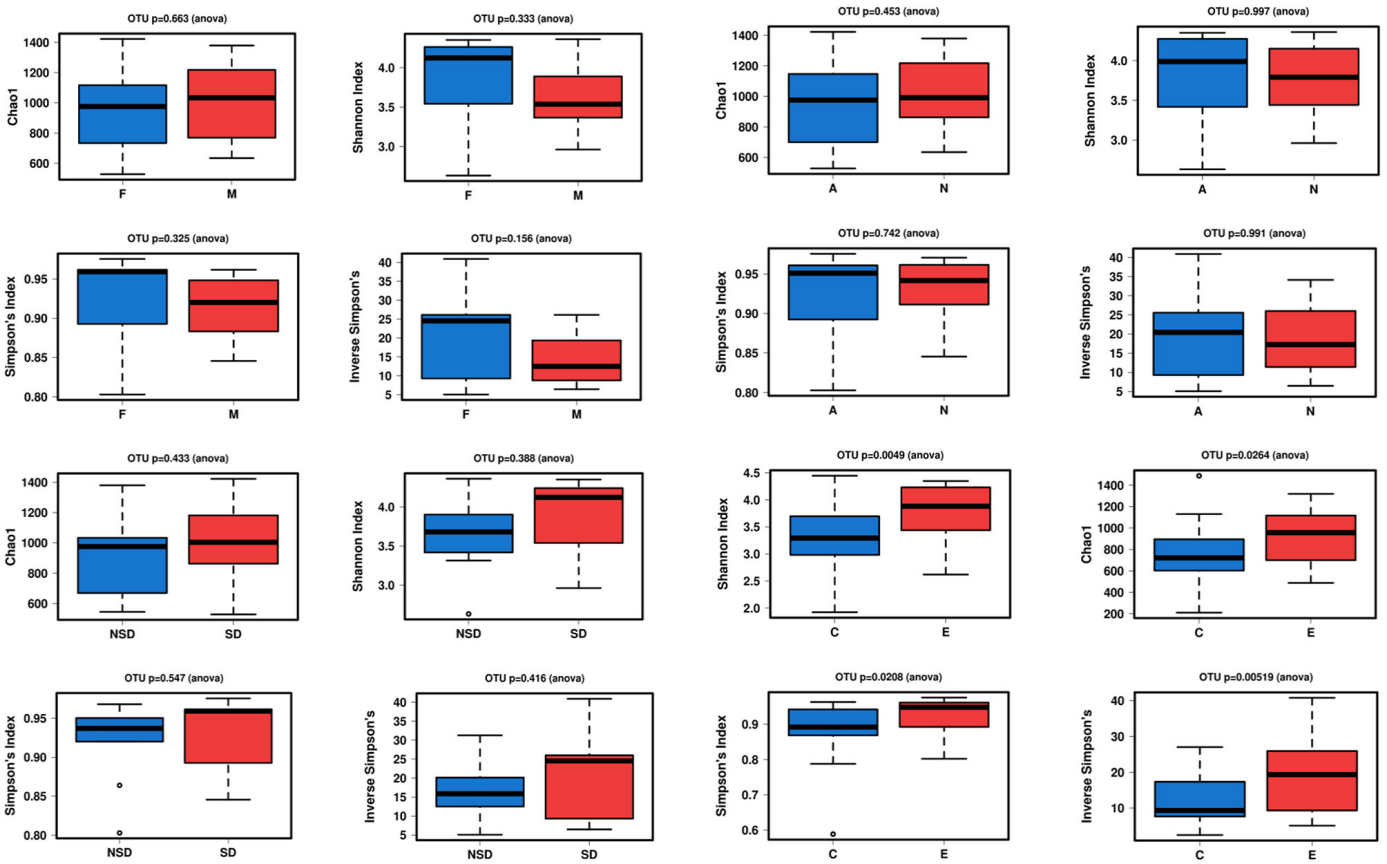
Alpha diversity between two groups with Chao1, Shannon, Simpson's, and inverse Simpson's indexes. (Significance is denoted by *p* < 0.05. The abnormal value is shown as “o”).

The alpha diversity indices of Chao1, Shannon, Simpson, and inverse Simpson were calculated to analyze the complexity of gut microbiome. Chao1 value can reflect the species richness of the community. Shannon, Simpson value can reflect both the richness and evenness of all the samples. In Figure 1, the alpha diversity of NERD group was higher than HC, which showed significant difference (*P* < 0.05). The results showed that species complexity of gut microbiome in NERD patients was significantly higher than that of the healthy control group. In addition, there was no significant difference between other groups with respect to the alpha diversity, indicating that the complexity of the bacterial communities between them were similar.

### Beta Diversity Analysis Principal Co-ordinates Analysis (PCoA)

**Figure 2:**
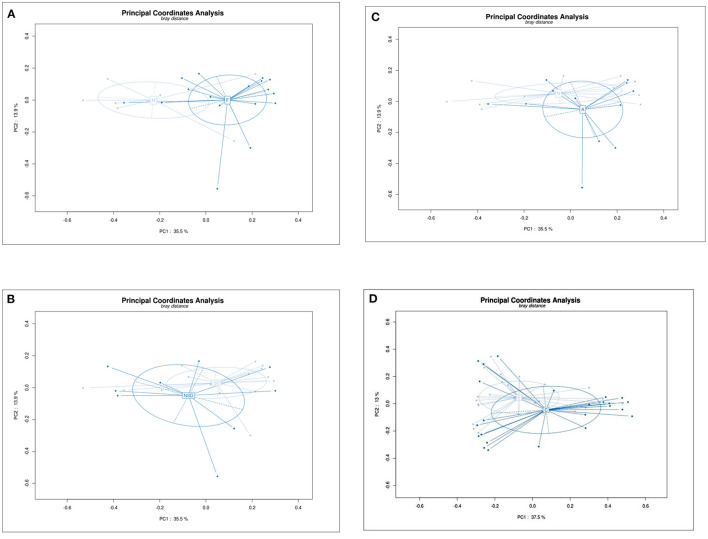
Principal Co-ordinates Analysis (PCoA) plot showing beta diversity among samples based on the OTU abundance. Each dot represents a sample, and different color depth represents a group (marked in the middle of circle). PC1 is the principal coordinate component causing the largest difference in samples, PC2 was next, with an explanatory value shown in axis. **(A)** Group F and M, PC1 (35.5%) and PC2 (13.9%), *p* = 0.09. **(B)** Group NSD and SD, PC1 (35.5%) and PC2 (13.9%), *p* = 0.531. **(C)** Group N and A, PC1 (35.5%) and PC2 (13.9%), *p* = 0.444. **(D)** Group C and E, PC1 (37.5%) and PC2 (13%), *p* = 0.0.026.

Beta Diversity Analysis Principal Co-ordinates Analysis (PCoA) is used to display differences among samples. If the two samples are close together, the species composition of the two samples is similar. As shown in Figure 2, PCoA revealed a separation in bacterial community composition between group C and group E using the first two principal component scores of PC1 (37.5%) and PC2 (13%), which was significantly different (*P* = 0.026). The beta-diversity was also significantly different between male and female patients (*P* = 0.009). No significant differences were found between other groups.

### Relative Abundance of Taxa Species Within Samples at the Level of Phylum, Class, Order, Family, and Genus Respectively

**Figure 3:**
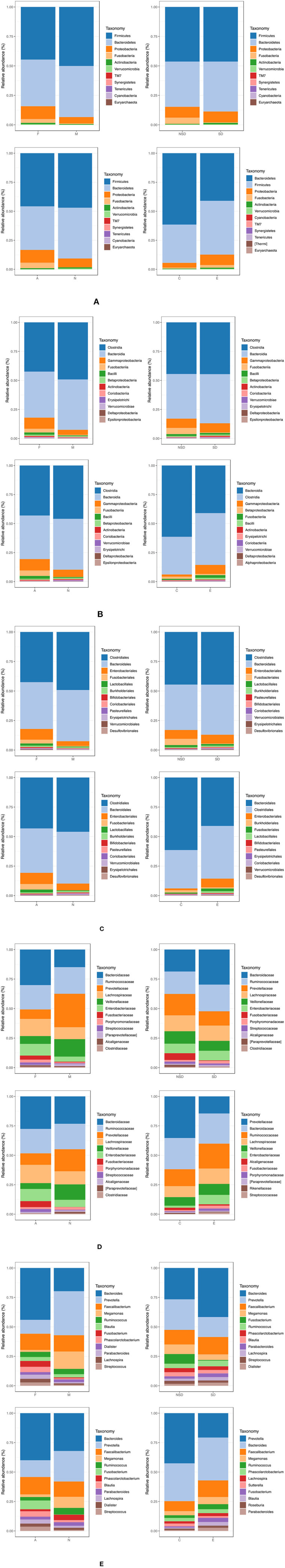
Bar graphs show taxonomic summary comparing relative abundance of taxa species within samples at the level of phylum, class, order, family, genus, respectively. (Groups: F, females, M, males; NSD, non-sleep-disorder, SD, sleep-disorder; A, NERD patients with somatoform disorders, N, NERD patients without somatoform disorders; E, experimental group including all NERD patients, C, control group). **(A)** Phylum, most abundant, **(B)** Class, most abundant, **(C)** Order, most abundant, **(D)** Family, most abundant, **(E)** Genus, most abundant.

We mainly analysed the microbial composition of groups at the phylum and genus level in detail, but we would not elaborate on the class, order and family level (Figures 3B–D).

*The Microbial Composition of Groups at the Phylum Level* (Figure 3A). In summary, the groups mainly contain 6 kinds of bacteria at the phylum level: Firmicutes, Bacteroidetes, Proteobacteria, Fusobacteria, Actinobacteria, and Verrucomicrobia. The majority of them are Firmicutes and Bacteroidetes although the ratio is different in groups. The proportion of other bacteria is minimal, such as TM7, and Synergistetes. It was found that Firmicutes increased and Bacteroidetes decreased in group E compared with group C. The similar ratio change of Bacteroidetes/Firmicutes also happened between groups N and A.

*The Microbial Composition of Groups at the Genus Level* (Figure 3E). The group F/M, group NSD/SD, and group A/N all contain 12 kinds of bacteria at the level of Genus: Bacteroides, Prevotella, Faecalibacterium, Megamonas, Ruminococcus, Blautia, Fusobacterium, Phascolarctobacterium, Dialister, Parabacteroides, Lachnospira, and Streptococcus. The group C and group E contain 12 kinds of bacteria at the level of Genus: Prevotella, Bacteroides, Faecalibacterium, Megamonas, Ruminococcus, Phascolarctobacterium, Lachnospira Sutterella, Fusobacterium, Blautia, Roseburia, and Parabacteroides.

***The analysis compared different groups at different taxonomic levels of evolutionary*** ***trees (phylum, class, order, family, genus) to identify bacteria with significant*** ***differences*.**

**Figure 4:**
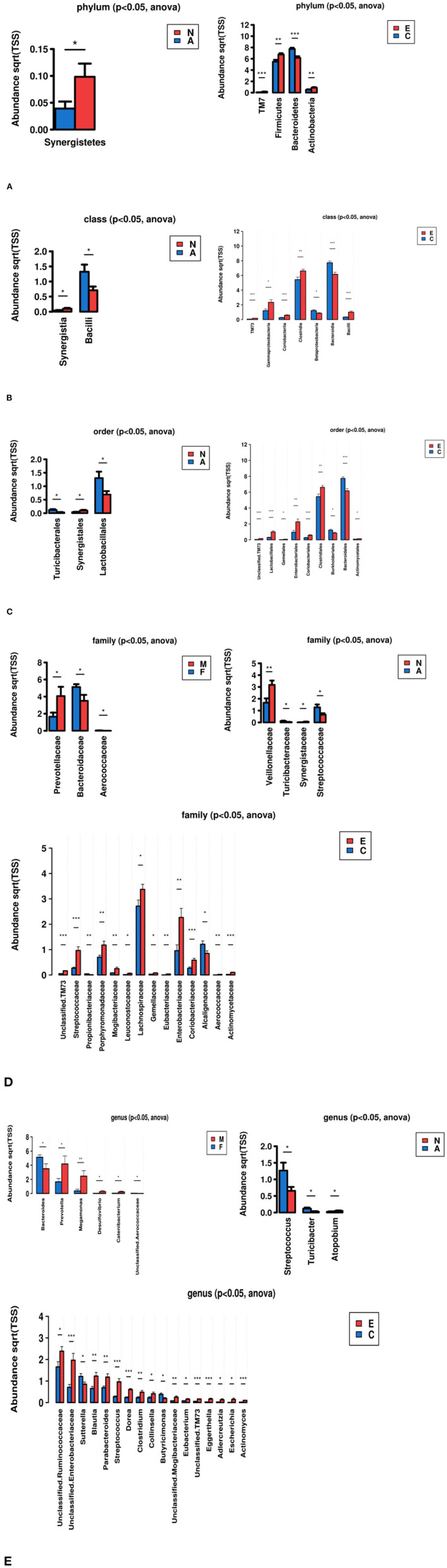
Identify bacteria with significant differences by comparative analysis between two groups (*p < 0.05, **p < 0.01, ***p < 0.001). **(A)** Phylum, **(B)** Class, **(C)** Order, **(D)** Family, **(E)** Genus.

These results in Figure 4 indicated that there were significant differences between group A and N, C and E. There was little difference between group F and M, NSD and SD. At the phylum level, *Firmicutes, TM7, Actinobacteria* were enriched in the group E compared with the healthy control group, while *Bacteroidetes* were enriched in the healthy controls. *Synergistetes* were enriched in group N compared with group A. At the genus level, Streptococcus, Turicibacter were richer in group A than group N, while Atopobium were less in group A than group N. Compared with healthy individuals, Unclassified.Ruminococcaceae, Unclassified.Enterobacteriaceae, Blautia, Parabacteroides, Streptococcus, Dorea, Clostridium, Collinsella, Unclassified.Mogibacteriaceae, Eubacterium, Unclassified.TM73, Eggerthella, Adlercreutzia, Escherichia, and Actinomyces were rich in the group E. Sutterella, Butyricimonas were enriched in group C compared with group E.

***The KEGG pathways for these functional genes of intestinal flora were involved in*** ***cellular synthesis, environmental information processing, genetic information processing*,** ***human diseases, metabolism, and others*.**

Among the pathways, some had especially significant difference, including Transporters, ABC transporters, Oxidative phosphorylation, Chaperones and folding catalysts, Carbon fixation pathway in prokaryotes, Energy metabolism, Porphyrin and chlorophyll metabolism, Citrate.cycle.TCA.cycle.

## Discussion

GERD includes non-erosive reflux disease (NERD), reflux esophagitis (RE), and Barrett's esophagus, and foreign epidemiological survey data showed that NERD accounts for about 50–70% of GERD (28). Patients with RE and NERD were often combined with mental and psychological abnormalities (29), which have attracted the attention of domestic and foreign scholars in recent years. For this group of patients, psychotherapy combined with medication can relieve clinical symptoms and improve quality of life to varying degrees in patients with NERD (12). In our study, we used several different questionnaires to evaluate the emotional state of patients. Finally, the psychological assessment results showed that above 60% of the subjects had emotional disorders including somatoform disorders, anxiety, and depression, most of them were mild to moderate, but the proportion was significantly higher than that of the general population, which provides similar evidence about a strong connection between the symptoms of NRED and emotional disorders as previous study showed (9, 30, 31). Among them, patients with somatoform disorders were accounting for 50%. Actually, we could boldly assume that some NERD patients were somehow equivalent to be part of the patients with somatoform disorders.

Estimated by previous research, about 10^14^ microorganisms reside in the adult gastrointestinal tract, which amounts to 10 times the number of cells in the human body. Most of them consist of bacteria from 500 to 1,000 different species that vary in stability, diversity, and number across different human populations, showing that the gut microbiome is more complex than those found in the mouth, esophagus, stomach, colon, vagina, and skin (32). However, very little data was found in the literature on the association between NERD and the gut microbiome (33). The present study also has provided some new contributions to the field of microbiome in patients with NERD. We have performed a study of the human gut microbiome rather than esophagus microbiome in NERD patients in which little prior knowledge was available. More importantly, the results confirmed that the diversity of gut flora was significantly different between NERD patients and the healthy controls. The gut microbiome of NERD patients was more complex, which may be associated with dysbacteriosis.

Among the gut microbiome, the Firmicutes and Bacteroidetes were the two dominant phylum (34, 35). Our research also got the same result in Figure 3. We found that Firmicutes increased and Bacteroidetes decreased in NERD patients and those with emotional disorders compared with healthy controls. Data from other GI illnesses, such as IBS, which are often accompanied by depressive symptoms, have revealed reduced Bacteroidetes and increased Firmicutes content in the fecal samples of patients with these disorders (36–38). In other aspects, the patients who suffered from heartburn and regurgitation for a long time may had a long-term anti-acid therapy. Long-term PPI use had been shown to decrease Bacteroides and increase Firmicutes in the gut, and meanwhile increase the risk of SIBO (small intestinal bacterial overgrowth) (39). When SIBO happened, food starch, polysaccharides, and fibers fermented to produce a large amount of gas, which increased abdominal pressure and affected gastric emptying, then aggravated regurgitation (40).

Furthermore, the intestinal flora was divided into three categories: beneficial bacteria, conditional pathogenic bacteria, and pathogenic bacteria. Conditional pathogenic bacteria, such as Enterobacteriaceae and Enterococcus, played an adverse role when the homeostasis of the body was broken. The results showed that Unclassified.Ruminococcaceae, Unclassified.Enterobacteriaceae, Blautia, Parabacteroides, Streptococcus, Dorea, Clostridium, Collinsella, Unclassified.Mogibacteriaceae, Eubacterium, Unclassified.TM73, Eggerthella, Adlercreutzia, Escherichia, and Actinomyces were rich in NERD patients compared with healthy individuals. The patients with somatoform disorders also had more Streptococcus but less Synergistetes. To some extent, NERD was also considered as a kind of inflammatory diseases. Bacteria such as Streptococcus, which are present in the human oral cavity, throat, and nasal cavity, increased in the intestine, implying that bacterial translocation, as well as enteric infections, may have occurred (41). Synergistetes was widely found in nature and had a high detection rate in oral diseases. Though it was found in gut microbiome, its proportion was very small and was meaningless. An up-regulation of the abundance of Ruminococcaceae can lead to degradation of the intestinal mucosa, instability of the intestinal barrier, and mild mucosal immune disorders (42). TM7 possibly played a role of inflammatory promoters in inflammatory gastrointestinal diseases. As for other microflora, there were few studies to clarify its specific function and correlation with NERD.

Overall, in recent years, the microbiome-brain-gut axis is fast becoming a topic of interest among researchers, which is vital for maintaining homeostasis, containing neural, humoral, immune, and metabolic pathways. This complex network of communication between the gut microbiota and the brain encompasses the CNS, the autonomic nervous system (ANS), the enteric nervous system (ENS), and the neuroendocrine and neuroimmune systems (32, 43). The dysregulation of microbiome-brain-gut axis has been implicated in various disease states. Current studies have shown that the diversity and types of intestinal flora are associated with anxiety and depression. Our study indicates that NERD patients with psychiatric disorders, especially with somatization disorder, had association with gut microbiome, considering that the microbial community residing in the gut plays an important role in the development of various aspects of brain function including anxiety, mood, cognition, and more recently in sociability.

Exactly, we predicted that microbiome in NERD patients played a role in the pathways of multiple substances metabolism, energy metabolism, Transporters, oxidative phosphorylation, Citrate.cycle.TCA.cycle, and so on, which perhaps effected the function of microbiome-brain-gut axis. Moreover, it was reported ATP-binding cassette (ABC) transporters could confer multidrug resistance by active efflux of intracellular drugs (44). In contact with NERD patients, we could consider that flora disorder may affect drug efficacy.

**Figure 5:**
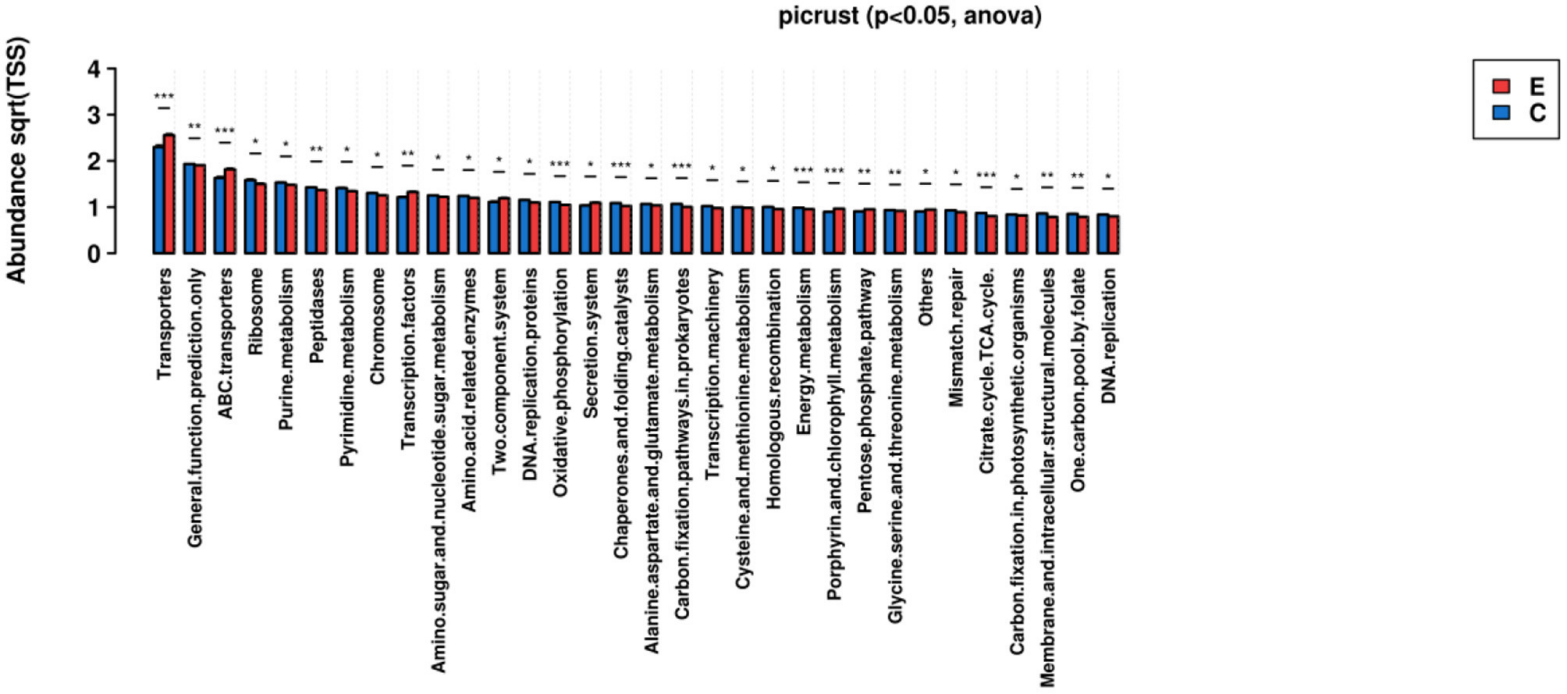
KEGG Ortholog functional profiles of microbial communities were predicted by PICRUSt (*p < 0.05, **p < 0.01, ***p < 0.001).

While we have identified some significant results, our study has certain limitations. First, the composition of the changing microbiome is distinct at different milestones of life. We cannot precisely identify the relationship of early life changes in stool microbiome to what is found in these patients as adults. Second, middle-aged patients were more likely to be enrolled, while young people often refused to participate in our study because of their individual considerations. This led to fewer young patients in the study and the results may be biased while considering all ages. Third, clinical data is limited by the small sample size although the sequencing depth and data amount of the samples are sufficient. The difference of intestinal flora among the groups in our research did not fully represent the composition of intestinal flora in the NERD patients. As we got the results shown in the Figure 5, there was little difference between female and male, patients with or without sleep disorder, which was probably caused by inadequate sample size. In the future, we would expand the sample size for further confirmation. Fourth, we limited our analyses to stool samples as we were interested in the association of gut microbiome rather than the esophageal microbiome as the former has already been reported, which could be divided into two categories, while type II may affect the occurrence of NERD by changing the speed of gastric emptying (45). We would prefer to perform duplications on drug-naïve subjects and compare alterations in NERD patients before and after pharmacotherapy. This present study provides some crucial background knowledge. Finally, it still needs to be further assessed whether our data support the use of probiotics-based interventions as adjuncts to current therapeutic strategies in NERD (46).

## Data Availability Statement

The datasets presented in this article are not readily available because it contains confidential personal information and data. Requests to access the datasets should be directed to the corresponding author.

## Ethics Statement

The study was approved by the Ethics Committees of Peking University Shenzhen Hospital, and all the patients signed the informed consent forms.

## Author Contributions

FY: conceptualization, methodology, investigation, formal analysis, visualization, writing original draft, and revising. X-hX: methodology, investigation, writing – review, and editing. XL: conceptualization, resources, data curation, and writing – review. H-nL: formal analysis and data curation. BZ: conceptualization, resources, supervision, validation, writing – review, and editing. All authors contributed to the article and approved the submitted version.

## Conflict of Interest

The authors declare that the research was conducted in the absence of any commercial or financial relationships that could be construed as a potential conflict of interest.

## Publisher's Note

All claims expressed in this article are solely those of the authors and do not necessarily represent those of their affiliated organizations, or those of the publisher, the editors and the reviewers. Any product that may be evaluated in this article, or claim that may be made by its manufacturer, is not guaranteed or endorsed by the publisher.
