# Supplementary figures and images for: Analysis of Psychological and Gut Microbiome Characteristics in Patients With Non-erosive Reflux Disease
Source: Front Psychiatry. 2022 Jan 13;12:741049. doi: 10.3389/fpsyt.2021.741049 (PMC8793911; doi:10.3389/fpsyt.2021.741049)

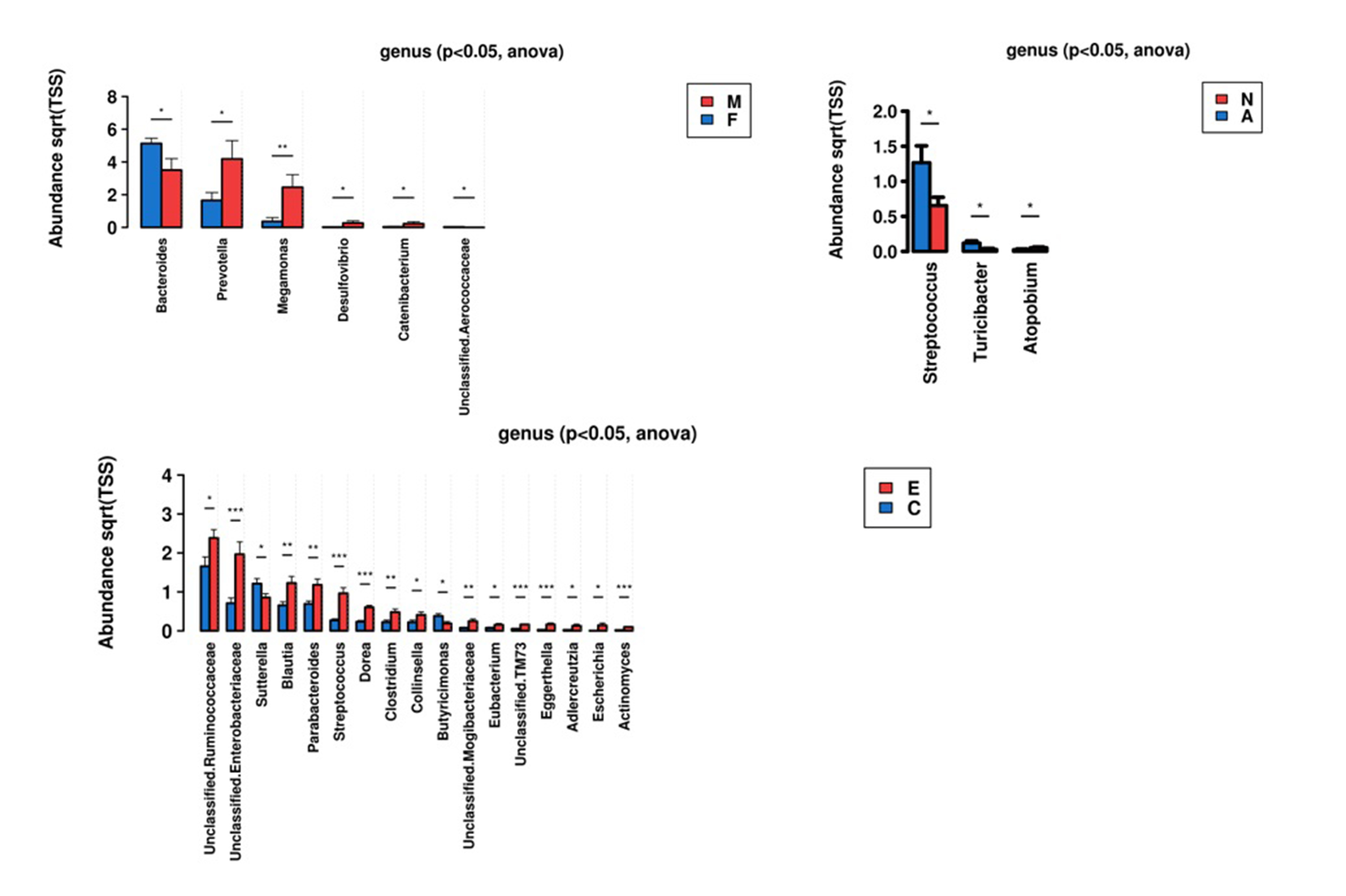

Supplement: Supplementary file 1 [file Image_1.JPEG]

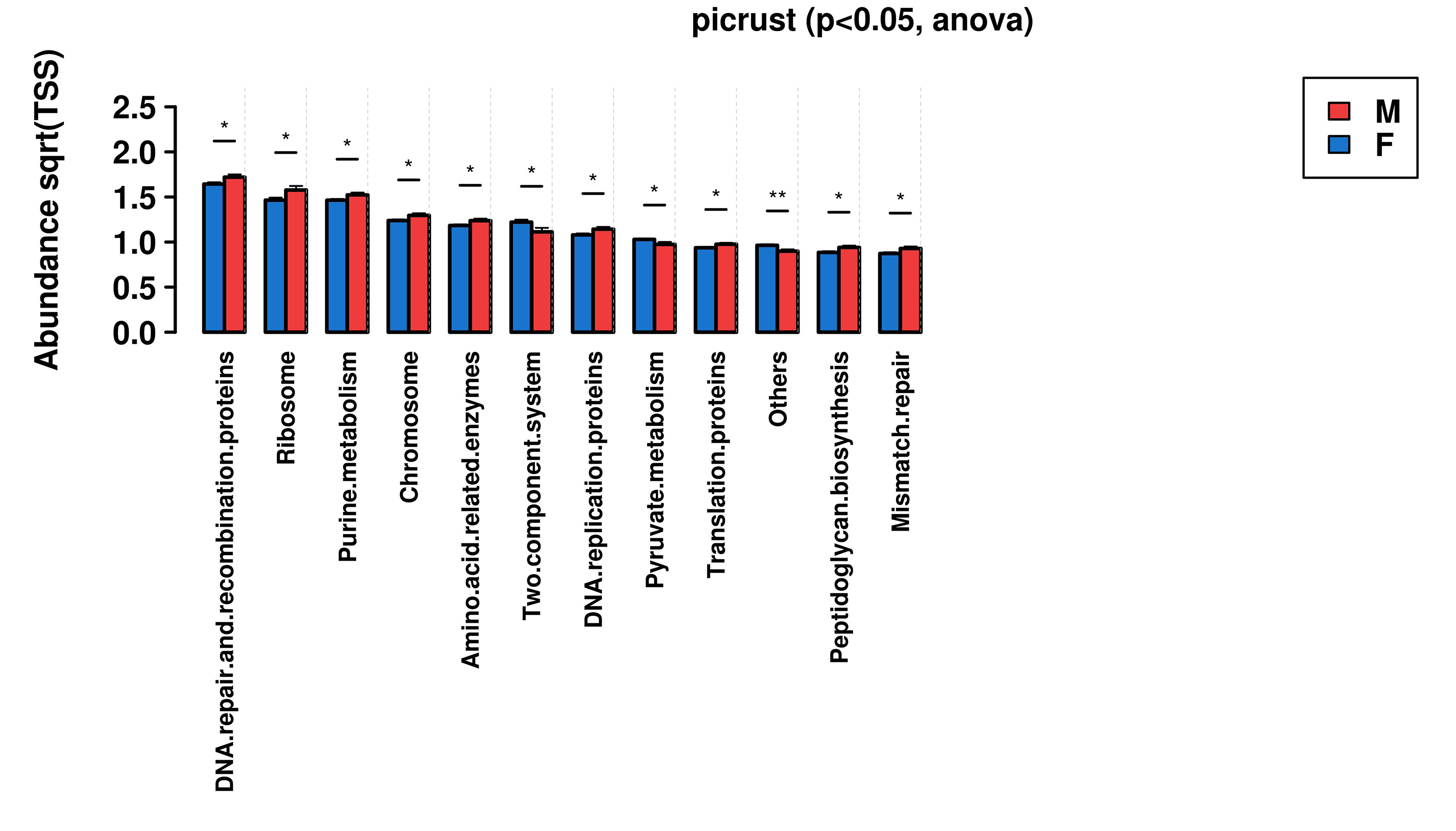

Supplement: Supplementary file 2 [file Image_2.JPEG]

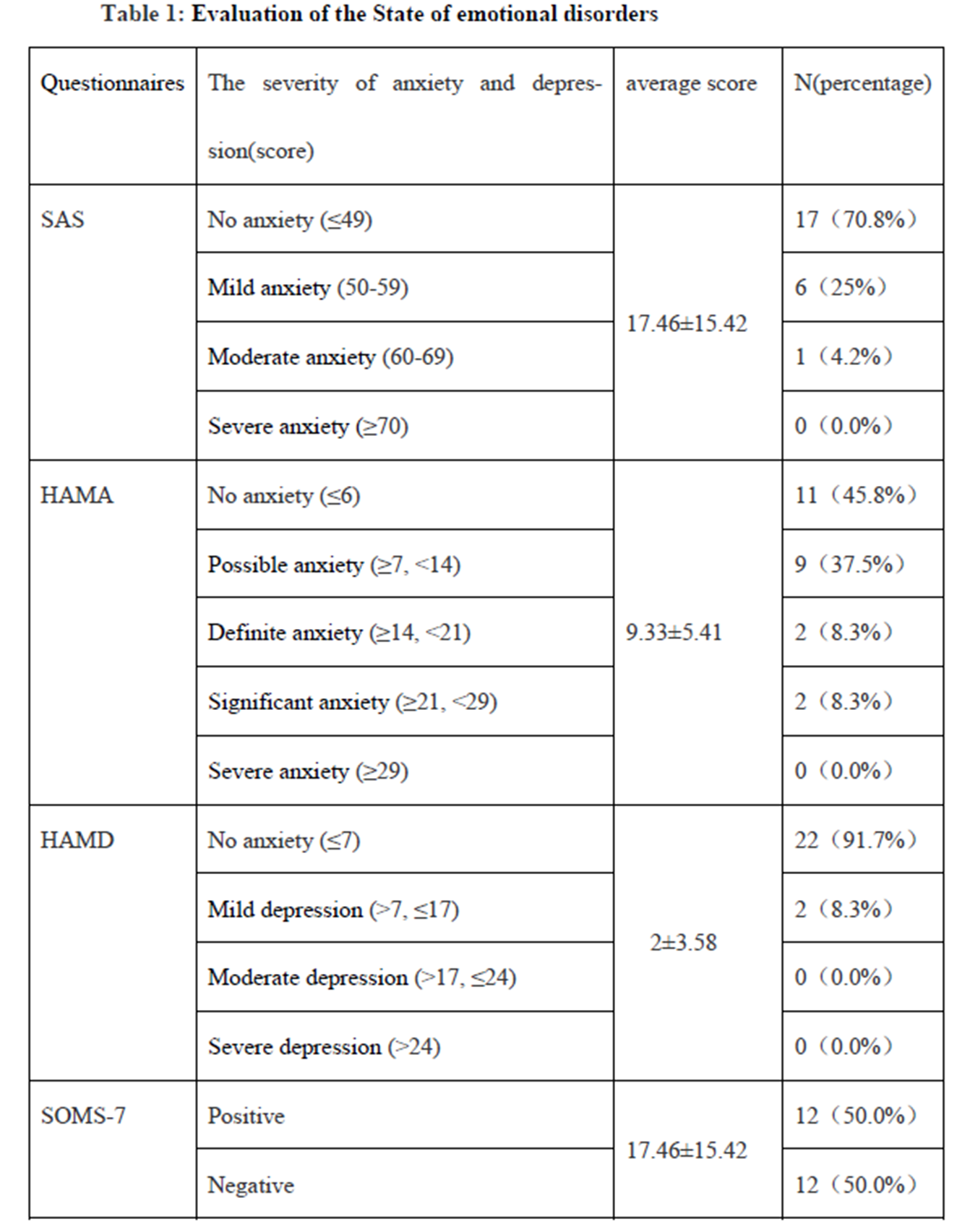

Supplement: Supplementary file 3 [file Image_3.PNG]
